# Better Epitope Discovery, Precision Immune Engineering, and Accelerated Vaccine Design Using Immunoinformatics Tools

**DOI:** 10.3389/fimmu.2020.00442

**Published:** 2020-04-07

**Authors:** Anne S. De Groot, Leonard Moise, Frances Terry, Andres H. Gutierrez, Pooja Hindocha, Guilhem Richard, Daniel Fredric Hoft, Ted M. Ross, Amy R. Noe, Yoshimasa Takahashi, Vinayaka Kotraiah, Sarah E. Silk, Carolyn M. Nielsen, Angela M. Minassian, Rebecca Ashfield, Matt Ardito, Simon J. Draper, William D. Martin

**Affiliations:** ^1^EpiVax, Inc., Providence, RI, United States; ^2^Institute for Immunology and Informatics, Providence, RI, United States; ^3^EpiVax Oncology, Inc., New York, NY, United States; ^4^Departments of Molecular Microbiology & Immunology and Internal Medicine, Division of Infectious Diseases, Allergy & Immunology, Saint Louis University, St. Louis, MO, United States; ^5^Center for Vaccines and Immunology, University of Georgia, Athens, GA, United States; ^6^Leidos Life Sciences, Frederick, MD, United States; ^7^National Institute of Infectious Diseases, Tokyo, Japan; ^8^Jenner Institute, University of Oxford, Oxford, United Kingdom

**Keywords:** bioinformatics, immunoinformatics, vaccines, EpiMatrix, ClustiMer, JanusMatrix, T cell epitope, Treg epitope

## Abstract

Computational vaccinology includes epitope mapping, antigen selection, and immunogen design using computational tools. Tools that facilitate the *in silico* prediction of immune response to biothreats, emerging infectious diseases, and cancers can accelerate the design of novel and next generation vaccines and their delivery to the clinic. Over the past 20 years, vaccinologists, bioinformatics experts, and advanced programmers based in Providence, Rhode Island, USA have advanced the development of an integrated toolkit for vaccine design called iVAX, that is secure and user-accessible by internet. This integrated set of immunoinformatic tools comprises algorithms for scoring and triaging candidate antigens, selecting immunogenic and conserved T cell epitopes, re-engineering or eliminating regulatory T cell epitopes, and re-designing antigens to induce immunogenicity and protection against disease for humans and livestock. Commercial and academic applications of iVAX have included identifying immunogenic T cell epitopes in the development of a T-cell based human multi-epitope Q fever vaccine, designing novel influenza vaccines, identifying cross-conserved T cell epitopes for a malaria vaccine, and analyzing immune responses in clinical vaccine studies. Animal vaccine applications to date have included viral infections of pigs such as swine influenza A, PCV2, and African Swine Fever. “Rapid-Fire” applications for biodefense have included a demonstration project for Lassa Fever and Q fever. As recent infectious disease outbreaks underscore the significance of vaccine-driven preparedness, the integrated set of tools available on the iVAX toolkit stand ready to help vaccine developers deliver genome-derived, epitope-driven vaccines.

## Introduction

Over the past 20 years, academic researchers and commercial companies have developed immunoinformatics tools to discover the T cell epitope “triggers” that activate the immune system and have applied these tools to vaccine design. The pace of algorithm development for discovering epitopes and designing vaccines has recently accelerated, due to renewed interest in epitope-based vaccines generated by the introduction of immune checkpoint inhibitors to the field of oncology (1) and the emergence of new pathogens such as nCOV-2019 (SARS-CoV-2).

This review will describe vaccine design tools developed by the authors and their application to epitope discovery, antigen engineering, and immunology research in wide-ranging collaborations, as illustrated in several case studies. Starting with T cell epitope discovery, we illustrate how T cell epitopes are relevant to vaccine safety, efficacy, antigen characterization, antigen engineering, and vaccine design. We believe that T cell epitopes deserve greater focus in vaccine development, because even in the absence of effective antibody response, T cell epitopes are important drivers of immune defense against pathogens and may also facilitate their escape from immune defense. Specifically, new information on cross-conservation between pathogen T cell epitopes and the human genome (and microbiome) is emerging, and has important implications for vaccine design. For example, “memory” of cross-conserved T cell epitopes has been defined as a key contributor to the strength of protection generated by vaccines (2–4). These discoveries are coming to light through the application of new tools that examine T cell epitopes and their role in vaccines, using the power of immunoinformatics.

**Figure 1:**
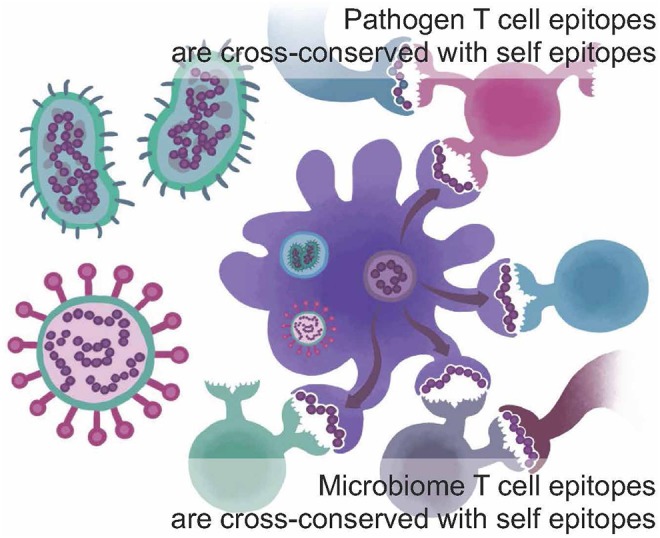
Immune camouflage. T cell epitopes found in the sequences of viruses, bacteria, and parasites and/or in the human microbiome may be conserved with highly prevalent T cell epitopes found in self-proteins from the human genome; cross-conservation may be observed at the TCR face for peptides that bind to the same HLA but do not have identical sequences. This may enable a pathogen to trigger “ignorance” or active tolerance from T cells that have been trained on self-epitopes. Adapted from Moise et al. (5).

One of the newest tools described here addresses the concept of immune camouflage (Figure 1). Beginning in 2013, we observed that the TCR face of epitopes presented by some pathogens contains patterns of amino acids that are identical to T cell epitopes that bind to the same HLA alleles and are highly prevalent in the human proteome (5, 6). We hypothesized that the role of these “human-like” T cell epitopes may be to tolerize against a pathogen, by activating self-reactive Tregs that suppress immune response (7). We also observed that immune camouflage is more likely to be present in pathogens that have the capacity to modify antigens (8, 9). We have generated evidence that this concept is also relevant to cancer vaccines and could improve the safety and efficacy of such vaccines (10).

After validating the immune camouflage hypothesis in retrospective (5) and prospective studies (4–10), we developed a new tool for iVAX called **JanusMatrix** that triages human-like epitopes from others. We highlighted the important role of human-like T cell epitopes in the abrogation of effective vaccine response. Furthermore, we used a tool called **OptiMatrix** to design **immune-engineered** vaccines that enhance immunogenicity by removing human-like epitopes (11). In other iVAX toolkit advances, to be described here, we explored the HLA-restricted or “personal” nature of immune response to novel antigens and developed specific tools to address these types of evaluations, called **iTEM** (individualized T cell epitope measure) and **EpiCC** (epitope content comparison), respectively. In the sections to follow, we will describe the newest iVAX tools, illustrate the application of the tools to current vaccine development programs, and highlight recent collaborations related to the development of vaccines against a range of human and animal pathogens.

## Methods: New Immunoinformatics Tools and the iVAX Toolkit

Computational vaccine design, also known as computational vaccinology, encompasses epitope mapping, antigen selection, and immunogen design using computational tools. *In silico* prediction of immune response to emerging infectious diseases and cancers can accelerate the design of novel and next generation vaccines. The iVAX toolkit is an integrated set of immunoinformatics algorithms that has been in development since 1998. It comprises a suite of immunoinformatics algorithms for triaging candidate antigens, selecting immunogenic and conserved T cell epitopes, eliminating potential regulatory T cell epitopes, and optimizing antigens for immunogenicity and protection against disease. While aspects of the toolkit have been published [see (12–14)], as of 2015, the iVAX toolkit has been significantly upgraded, new tools have been integrated and validations of the new tools have been published. Here we will focus the newest tools and provide illustrated examples of iVAX applications.

### Overview of the iVAX Toolkit

**Figure 2:**
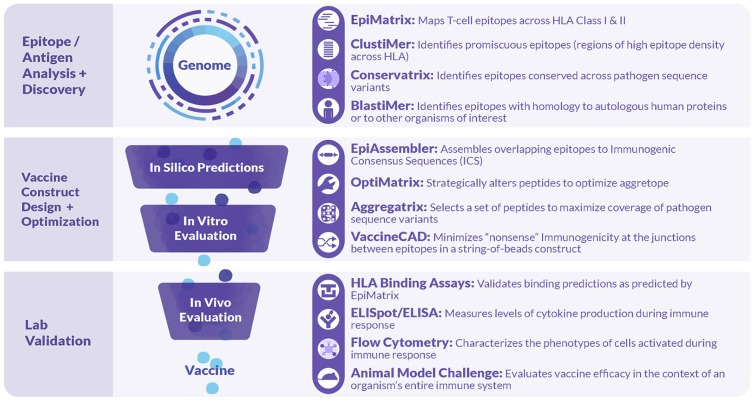
Tools comprised in the iVAX Toolkit and laboratory tools used in validation studies. The iVAX toolkit is an on-line, secure access toolkit that provides individualized sites for academic and commercial users. Sequences can be uploaded and analyzed for Class I and Class II T cell epitopes using EpiMatrix and searched for clusters of epitopes (promiscuous epitopes) using ClustiMer. Tools such as Conservatrix define cross-strain conserved epitopes and JanusMatrix identifies T cell epitopes that induce anergy or active tolerance. These algorithms, and others such as VaxCAD and iTEM are integrated into the secure-access, cloud-based toolkit. Beyond the *in silico* analysis phase, vaccine design usually proceeds to *in vitro* and *in vivo* validation.

As illustrated in Figure 2, iVAX incorporates a large number of tools that can be used sequentially or individually to manipulate information derived from the core T cell epitope mapping tool, EpiMatrix (15). Tools such as the **Conservatrix, EpiMatrix, ClustiMer**, and **EpiAssembler** algorithms have been described in great detail previously (14). Newer tools include the **VaxCAD** algorithm (16) that creates string-of-beads vaccine designs while minimizing deleterious junctional epitopes that may be created in the process of linking one epitope to another. Additional tools that have been integrated into the website since 2015 include **JanusMatrix**, a specially tailored homology analysis tool that examines pathogen/host sequence similarity at the MHC:TCR interface for any given peptide, and predicts potentially cross-conserved epitopes, allowing candidate sequences with potential host cross-conservation (at the TCR face) to be preferentially excluded from vaccine constructs, and **iTEM**, which enables the analysis of an individual's immune responses to vaccine antigens according to their HLA haplotype. The latter two tools have also been integrated into a separate pipeline for *personalized* cancer vaccine design called Ancer, that has been licensed to EpiVax Oncology, an investor-backed spin out of EpiVax.

### Assessing Protein Antigens for Immunogenic Potential Using EpiMatrix

Every vaccine design project begins with an analysis for T cell epitope content. This search for class I HLA ligands or class II HLA ligands (and putative epitopes) is performed using EpiMatrix (15). Antigen sequences obtained from databases such as GenBank or UniProt are input in FASTA format. In theory, there is no need to limit to the number of input sequences, and entire host genomes (e.g., *Mycobacterium tuberculosis, Burkholderia mallei and pseudomallei, and Coxiella burnetii*) have in fact been analyzed (17, 18).

To perform an EpiMatrix epitope-mapping analysis, each input sequence is first parsed into overlapping 9-mer frames (and 10-mer frames for Class I). Parsed 9- and 10-mers are then evaluated for patterns that match known HLA-binding preferences for a panel of nine common “supertype” Class II HLA alleles or six Class I supertypes alleles using EpiMatrix (19). These alleles are selected both because they are relatively common within the human population, and relatively distinct from each other, and, each supertype has HLA binding preferences that are functionally equivalent to, or nearly equivalent to, many additional family member alleles. Taken collectively, the nine HLA Class II supertype alleles, along with their respective family members, cover well-over 95% of most HLA types present in human population groups (20), and the six supertype alleles, along with their respective family members, cover over 98% of the human population groups including East Asian populations (21).

Even though antigen analysis is generally performed at the population level, there are many instances when a more precise HLA-by-HLA analysis may be required. Over the past 5 years, EpiMatrix has been updated to include many more HLA alleles than the standard six Class I “supertypes” and Class II “archetypes” (2,217 Class I and 612 Class II alleles are now available) as well as murine (H-2K, H-2D, I-A, and I-E haplotypes for Balb/C and C57Bl/6) and epitope prediction for swine SLA alleles (22) (15 Class I and 8 Class II alleles are now available). This capacity to evaluate for a set of HLA enables personalized predictions, making the toolkit useful for evaluating individual subject responses to vaccine antigens, or for exploring HLA-specific vulnerabilities to pathogen escape.

**Figure 3:**
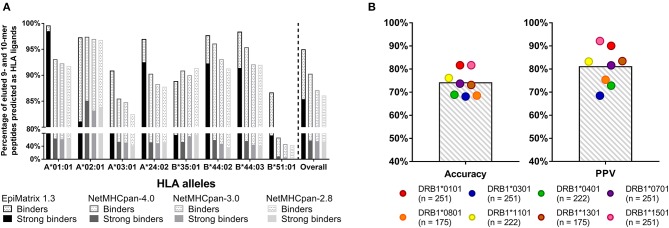
Recent validation studies: **(A)** CD8 T cell epitopes. Retrospective analysis of eluted peptide dataset published by Abelin et al. (23) 95% of eluted 9- and 10-mers are predicted to bind to HLA according to EpiMatrix, many of which are predicted strong binders (solid bars). Only ~88% of ligands were accurately recalled by various versions of NetMHCpan. EpiMatrix also identifies the majority of the eluted ligands as high affinity binders (85% of all eluted peptides, solid bars), whereas only up to 58% of ligands were identified as high affinity binders (IC50 ≤ 50 nM) by NetMHCpan. **(B)** CD4 T cell epitopes. Prospectively validation of EpiMatrix selections in *in vitro* HLA binding assays: EpiMatrix HLA class II predictions are 74% accurate when tested in *in vitro* HLA binding assays, with an average observed Positive Predictive Value (PPV) of 81%. The number of peptides tested for each HLA allele is shown in parenthesis. Selected peptides were published in reference 23 and some are unpublished.

The data used to generate EpiMatrix matrix-based predictors has been described previously (15). Sources for the development of the tools include the Immune Epitope Database (IEDB), where, for example, 25,764 unique T cell epitopes, HLA ligands, and eluted peptides have been collected for the human HLA A^*^0101 allele. Each of the more than 39 class I matrices and 42 class II matrices are based on similarly large datasets. From this starting point, we apply internal algorithms for cleaning the database to downselect the final list of epitopes that inform our matrices. Careful curation of high-quality binders and removal of low-quality binders enables EpiMatrix to perform better (on average) than other tools, as illustrated in a recent head-to-head comparisons of published HLA class I ligands (Figure 3).

Figure 3A shows a recent comparison of EpiMatrix score of eluted peptides to those generated by other publicly available tools (NetMHCpan) that was made possible by the publication of a large set of eluted peptides (23). Figure 3B shows recent HLA binding studies for HLA class II-restricted epitopes, performed at EpiVax (published and unpublished). Additional T cell epitope and HLA binding validation studies have been published in the course of grant-funded research collaborations, describing T cell immune responses to predicted epitopes *in vitro* using human lymphocytes [see (4, 24–32)] and also in prospective, *in vivo* immunogenicity and vaccine efficacy studies in murine models [see (10, 18, 33–38)].

### Scaling Antigens for Their Potential for Immunogenicity

#### T Cell Epitope Content

In order to select antigens for vaccine design, it is important to consider the overall potential for immunogenicity, which is directly related to cytotoxic T cell (CTL) or T helper (Th) T cell epitope content. Even though the effect of adjuvants and the innate immune system is critically important for immunogenicity, these “danger signals” are unlikely to be effective if there are no T cell epitopes for the activated immune system to react to. Thus, we have hypothesized that the greater the concentration of HLA ligands and putative T cell epitopes that are contained in an antigen, the more likely it will induce an immune response.

This hypothesis has generally been supported in prospective studies, with the caveat that correction of the total T cell epitope count for the presence of Treg epitopes is likely to be important, but methods for performing that analysis on a large scale have not yet been extensively validated in prospective studies, and thresholds for cross-conservation have not been extensively tested (for whole antigens), and thus our group prefers to evaluate antigens for putative Treg epitopes on a case-by-case basis.

#### Protein Immunogenicity Scale

T cell epitope concentration can be expressed as an overall EpiMatrix score called the **EpiMatrix Protein Score**, which is the difference between the number of predicted T cell epitopes expected in a random protein sequence and the number of putative epitopes predicted by the EpiMatrix System in a given protein, normalized for length (per 1,000 amino acids). With the average number of T cell epitopes contained in 10,000 randomly generated protein sequences set to zero, highly immunogenic vaccine antigens score higher than 20 on this normalized scale.

### Comparing Antigens to the Human Proteins

Human proteins generally score lower than the randomly generated proteins on this scale. In a recently performed assessment of the class II HLA DR T cell epitope content of human proteins (39), the median score of the entire human proteome was found to be −9.05, suggesting that T cell epitopes, which could support deleterious autoimmune activity, tend to be present in lower than expected numbers. The median score of secreted proteins is even lower, −23.08, suggesting that proteins with a greater likelihood of uptake and presentation by APCs are further deimmunized.

**Figure 4:**
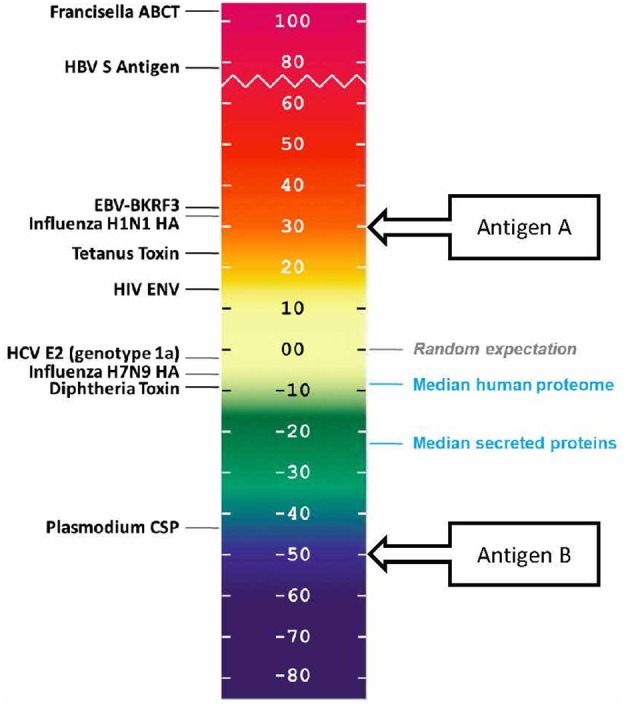
EpiMatrix immunogenicity scale, showing vaccine antigens, human proteins that have been reported to be immunogenic, and non-immunogenic antigens alongside the median scores for sets of proteins from the human genome. The vaccine antigen sequences were obtained from UniProt. The EpiMatrix immunogenicity scale is set to zero based on the median HLA DR score of a set of random protein sequences. Normalization of the HLA scoring enables direct comparison of median HLA DR scores to candidate antigens; for example, candidate vaccine antigen A would be preferred over candidate vaccine antigen B for inclusion in a vaccine designed to elicit T helper immune response and to drive humoral response.

In other words, it may be evolutionarily advantageous for the human proteome to be “deimmunized” with respect to T cell epitope content (as shown in blue, in Figure 4). We first described this concept in 2006, when we proposed that it may have been advantageous for self-proteins to be “deimmunized” to reduce the likelihood of auto-reactivity (40). A similar propensity has been observed for some, but not all human pathogens (8, 9).

### Regional Immunogenicity

The normalized **EpiMatrix Protein Score** of a given protein is a good proxy for immunogenicity, however, regional immunogenicity can also determine the immunogenic potential of a protein. T cell epitopes have been observed to cluster in regions of protein sequences. T cell epitope “clusters” are defined using **ClustiMer**, a cluster-finding algorithm in iVAX. Clusters can range from nine to roughly 25 amino acids in length and can contain anywhere from four to forty HLA binding motifs. T cell epitope clusters scoring above 10 are considered to have significant immunogenic potential. Some T cell epitope clusters contain a single 9-mer frame that may contain sequences that can bind to at least four different HLA alleles. iVAX denotes this feature as an “EpiBar” for its bar-like appearance in EpiMatrix reports.

**Figure 5:**
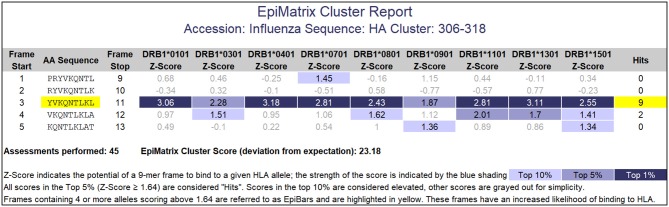
EpiMatrix Class II analysis. This analysis of an influenza hemagglutinin sequence shows a 9-mer frame that contains 7 top 1% hits and 2 top 5% hits for HLA DR alleles. This feature is called an EpiBar (Epitope bar) and is a feature of highly immunogenic epitopes. This analysis was performed using HLA DRB1 alleles.

Peptides containing promiscuously binding epitopes such as EpiBars can be very powerful immunogens. Examples include the well-known T cell epitopes used as controls for T cell assays (41), including Influenza Hemagglutinin 306–318 (Figure 5), Tetanus Toxin 825–850 and GAD65 557–567. We have observed that 100% of subjects exposed to either Tularemia or Vaccinia respond to T cell epitope pools containing between 20 and 50 promiscuous epitopes (25, 26).

Clusters are also important features of self-proteins and are often involved in auto-reactive T cell responses. Well-known “auto-epitopes” such as insulin peptide C23-A2 (LQPLALEGSLQKRGI) contain significant numbers of HLA DR binding motifs (42). To better define immunogenic epitopes, we have determined that an EpiMatrix Cluster score cut off of 10 (roughly defined as the number of significant HLA binding “hits” divided by sequence length) is a feature of highly immunogenic clusters.

Also in contrast with the general trend toward “deimmunization” observed for most of the proteins in the human genome, we found that Complement Factor 3 subunit D (C3d) has a higher concentration of T cell epitopes, which suggests that it may perform the important function of providing non-cognate help to drive humoral immune response when it binds to antigen and is taken up by B cells. This peptide is located in the previously defined P28 “adjuvant” region of C3d which is completely conserved across species (43, 44). Mutation of a single T-cell epitope in P28 resulted in significantly diminished adjuvant activity of the peptide in mice (45), which supports the hypothesis that the epitope activates autoreactive T-helper cells so as to bridge innate and adaptive immunity.

### JanusMatrix and Human-Like T Cell Epitopes

Although T cells possessing anti-self TCRs were previously thought likely to be eliminated in the thymus, evidence emerged showing that anti-self-immune response is also controlled by regulatory T cells recognizing the same antigens (46, 47). The phenotype of these regulatory T cells may be reinforced by repetitive re-exposure to their cognate self-antigens (48). Thus, human immune response to new antigens is shaped by previous experience in the thymus and by exposure-driven reinforcement in the course of immune system maturation.

In 2013, we observed that critical antigens from human pathogens contained T cell epitopes that are highly conserved with self-antigens, and postulated that these epitopes might induce “ignorance” or active tolerance to the antigen upon vaccination, resulting in “immune camouflage” (7). In retrospective studies, we determined that peptide epitopes that have homologs in the human proteome that have compatible, but not exactly matched, MHC binding anchors and exactly matched TCR-facing contours have the potential to be ignored (lack of immune response) or induce active tolerance (activate T cells that are immunosuppressive in bystander assays and/or exhibit cell surface markers that are consistent with T cells known to have a regulatory T cell phenotype). We developed the JanusMatrix tool so as to identify these human-like epitopes and have been exploring the impact of modifying and excluding these epitopes to improve the antigenicity of vaccines (49).

**Figure 6:**
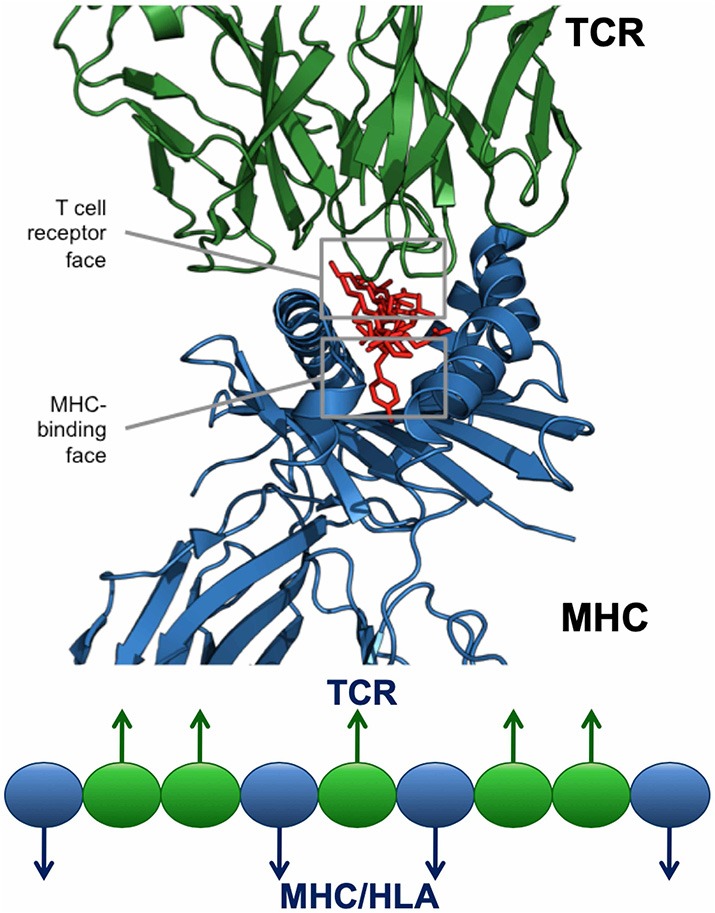
JanusMatrix and self-like epitopes. Each HLA ligand has two faces: the HLA-binding face (agretope, blue residues), and the TCR-interacting face (epitope, green residues). Predicted ligand with identical TCR epitopes and variant HLA-binding agretopes may stimulate cross reactive tolerizing or Treg responses, providing they bind to the same HLA allele.

For any given T cell epitope, JanusMatrix defines the amino acids that contact and bind the MHC molecule, and also identifies those amino acids that make contact with the TCR of responding T cells. For Class II restricted epitopes, positions 1, 4, 6, and 9 are assumed to make contact with the MHC and positions 2, 3, 5, 7, and 8 are assumed to be available to the TCR (Figure 6). The TCR facing residues of Class I epitopes varies from allele to allele.

The JanusMatrix algorithm then searches a reference database (which may be human, murine, or other organism), for similar epitopes, taking into consideration epitope conservation of the MHC-facing agretope and the TCR-facing epitope. Reference sequences with a compatible but perhaps not sequence-identical agretope (i.e., one that is predicted by EpiMatrix to bind the same HLA as the input peptide) and exactly matching the TCR contacts of the input peptide are returned. For a given EpiMatrix Score, a high JanusMatrix Homology Score suggests that T cells recognizing that epitope may exhibit a bias toward immune tolerance, a hypothesis that has been confirmed in retrospective and prospective studies (14). We use a cutoff of two (cross-conserved HLA-allele-specific epitopes averaged over the length of the sequence) for proteins that are not self, and of three for proteins that are 'self', to identify epitopes that are more likely to be tolerated or actively regulatory. This cutoff has been published (4).

In the first prospective test of this tool, we were able to identify T cell epitopes from hepatitis C virus (50) with high JanusMatrix Scores that induce regulatory T cells *in vitro* and suppress immune responses to other peptides. Specifically, JanusMatrix identified a promiscuous class II epitope in non-structural HCV protein p7 that exhibits homology at the TCR face with hundreds of human proteome-derived T cell epitopes and induces an increase in CD4^+^CD25^+^FoxP3^+^ Treg number and function in PBMC cultures derived from an HLA-diverse cohort of HCV-infected patients.

Additionally, we found similar human-like epitopes in emerging H7N9 influenza (6, 11). The ability of class II-restricted H7N9 epitopes to activate or modulate effector T cell responses was correlated with their degree of cross-conservation at the TCR-face with similar epitopes in the human proteome, as defined by JanusMatrix. These epitopes activate human CD4^+^CD25^high^CD39^+^FoxP3^+^ Tregs and suppress H7N9-specific effector T cell responses (6). Deletion of the Treg-inducing epitope in H7N9 HA produced an antigenically improved HA that stimulated a 6-fold increase in activated, effector CD4^+^ T cells over wild type H7-HA *in vitro*, and stimulated a 5-fold greater anti-H7-HA IgG titer and 20-fold greater anti-H7-HA B cell frequency over mice immunized with wild type protein in humanized mouse immunization (11).

In the biologics area, we have discovered conserved Treg-inducing epitopes (Tregitopes) in immunoglobulin G with TCR-face patterns found in several self-proteins. We went on to demonstrate that the Tregitope peptides bind to multiple MHC class II molecules, suppress effector T cell responses to co-delivered antigen, upregulate Treg-associated cytokines and chemokines, and induce antigen-specific CD4^+^CD25^+^FoxP3^+^ Tregs *in vitro* and *in vivo* (51, 52). While once considered novel, the idea that natural regulatory T cells can be engaged and activated by peptide epitopes possessing TCR-facing motifs commonly found within human proteins is now gaining traction (53) and concepts that are similar to JanusMatrix have also been described by other research groups (54, 55).

### Personalized, or Individualized T Cell Epitope Analysis (iTEM and J-iTEM)

We also recently determined that T cell epitopes that stimulate CD4 T helper cells in specific human subjects can be identified by immunoinformatic analysis to predict the antigenicity of individual epitopes or whole proteins, utilizing the patient's HLA type. To perform this analysis, we focus the prediction on patient-specific HLA alleles using the **individualized T cell epitope measure (iTEM)** tool (56) and correct for cross-conserved epitopes presented by the same HLA allele using JanusMatrix. The analysis that results from the combination of these two tools is now called **J-iTEM**. Case studies using this new combination of tools are provided below.

### Integrating PigMatrix Into iVAX for Animal Vaccine Development

We developed epitope prediction tools for swine and published the method used to develop the tools in 2013 (22); these tools can now be selected instead of human HLA-epitope prediction tools, enabling the entire iVAX toolkit platform to be applied to infectious disease affecting swine. This tool is currently being applied to the development of a universal influenza vaccine for pigs (57), and to prepare a vaccine for the eventual emergence of African Swine Fever (ASF) in the USA. Addressing another important issue that confronts hog farmers, we developed an immunoinformatic approach that matches existing vaccines with circulating strains to help farmers pick the best vaccine for their individual pork farm, that might mitigate against disease when vaccine-induced antibody does not protect. This tool is called EpiCC (58) for T Cell Epitope Content Comparison and is under evaluation by commercial vaccine companies for applications to a wide range of swine pathogens including PCV2. Higher EpiCC scores are thought to be associated with greater vaccine protection against challenge strains, and the first study of this method identified a “threshold of protection” that may determine whether a vaccine contains sufficient T cell epitope relatedness to provide cross-conserved immune protection against challenge strains.

## Results: Recent Case Studies using iVAX

### Development of CD8^+^ and CD4^+^ T Cell-Targeted Universal Influenza Vaccines

In a collaboration with Chris Eickhoff and Dan Hoft at Saint Louis University, we identified CD4^+^ and CD8^+^ T cell epitopes that are highly conserved in diverse influenza A strains that were shown to be immunogenic in humans expressing genetically diverse MHC (59). Proof-of-principle studies were conducted using novel vaccines incorporating these newly defined T cell epitopes in HLA transgenic mouse strains that respond to the same peptide/MHC combinations as human T cells. These vaccines elicited robust T cell responses and provided significant protection against diverse influenza A challenges, providing strong support for T cell-targeting universal influenza A vaccines. Efforts to develop a universal influenza vaccine are ongoing in collaborations with researchers in the United States (Saint Louis University, University of Georgia) and Europe.

### Predicting Immunogenic *Coxiella burnetii* T Helper Epitopes

Building on our experience with the “rapid fire” development of a vaccine for Lassa Fever (60), EpiMatrix and JanusMatrix were used to identify 50 promiscuous class II epitopes from *Coxiella burnetii* antigens that were tested in HLA binding assays and screened for immunogenicity in HLA-DR3 transgenic mice by colleagues at the Vaccines and Immunotherapy Center (VIC, Harvard) and Innatoss, Oss, the Netherlands (28, 29). Significant epitope-specific IFNγ responses were found for 11/50 peptides, all of which are predicted HLA-DRB1^*^0301 epitopes (Fisher's exact *p*-value: 0.023); all but one bound to DRB1^*^0301 *in vitro*. A vaccine featuring these epitopes is in development by a consortium based at the Vaccines and Immunotherapy Center (Harvard) and may eventually replace the existing vaccine (QVax) and/or the T cell epitopes may be used to develop diagnostic reagents (29).

### Optimized Selection of *Plasmodium falciparum* Circumsporozoite Protein T Helper Epitopes

Helper CD4^+^ T cells are central to development of protective immune responses to *P. falciparum* malaria, playing a key role in the B cell activation and maturation process. Working with Amy Noe and Vinayaka Kotraiah at Leidos, we used iVAX to predict and analyze HLA class II-restricted epitopes that would drive humoral immune responses through cognate T cell help, providing broad population coverage for a novel *P. falciparum* CSP protein (PfCSP) vaccine (61). In the course of a USAID-funded program, more than 450 PfCSP epitope sequence variants were analyzed and the optimal sequences (with highest cross-strain coverage) were selected for the next stage of vaccine development. We also noted high interstrain variability in CSP T helper epitopes and a tendency for the more highly conserved CSP epitopes to be more cross-conserved with the human genome (62).

### iTEM Analysis: Analyzing Individual Responses to Vaccines

The RH5 antigen is a highly conserved *P. falciparum* blood-stage antigen that was recently assessed in a Phase I clinical trial of controlled human malaria infection by Draper and colleagues at Oxford. We identified immunogenic (and non-human-like) epitopes in RH5 using iVAX and also analyzed the immunogenic potential of an RH5 overlapping peptide set used in immune recall studies. Using EpiMatrix, ClustiMer, JanusMatrix, and iTEM (Individualized T cell Epitope Measure) as well as an adaptation of JanusMatrix to iTEM (J-iTEM), we found T cell responses directly correlated with the presence of HLA-DR restricted ligands defined using EpiMatrix and the absence of human-like T cell epitopes. Peptides inducing positive responses had higher iTEM and J-iTEM scores (*p* < 0.05 and < 0.01, respectively) than negative peptides (63).

### Retrospective Analysis of Individual Responses to Peptides From Vaccinia Using iTEM

Class II T cell epitopes from vaccinia virus proteins were identified in IFNγ ELISpot assays using the PBMCs of smallpox vaccinees in a research study that used PBMC from clinical study volunteers (64). An overlapping peptide library of four vaccinia membrane proteins known to induce an immune response in vaccinated individuals was synthesized and tested in IFNγ ELISPOT assays using the PBMCs of 29 recent smallpox vaccine recipients. These T cell epitope sequences were retrospectively analyzed using EpiMatrix and JanusMatrix. We found that the peptides identified as T cell epitopes were predicted to bind to class II HLA supertype alleles more often than the remainder of the overlapping peptide library (*p* < 0.05) and that the peptides identified as class II T cell epitopes had lower JanusMatrix human homology scores than the remainder of the overlapping peptide library (*p* < 0.05). This is consistent with the identification of T cell epitopes as described by the publication, suggesting that *in silico* binding predictions correlated to T cell responses *in vitro*.

### Integrating PigMatrix Into iVAX for Animal Vaccine Development

In a proof of concept study that was recently published with Crystal Loving and colleagues at Iowa State, we used immunoinformatics tools to identify class I and II T cell epitopes highly conserved in seven representative strains of IAV in US swine and predicted to bind to prevalent Swine Leukocyte Antigen (SLA) alleles (57). The efficacy of an intradermal-delivered plasmid DNA vaccine composed of these epitopes against H1N1pdm09 challenge was compared to an intramuscular commercial inactivated whole virus vaccine and a heterologous prime-boost approach using both vaccines. Recently published results suggest the heterologous prime-boost approach using an epitope-driven DNA vaccine followed by an inactivated vaccine was effective against a homosubtypic challenge, illustrating the utility of immunoinformatic approaches to vaccine design (65).

### T Cell Epitope Content Predicts Vaccine-Related Protection Against Emerging Infectious Diseases

The EpiCC tool has been applied successfully to describe vaccine protection against challenge strains for influenza A (58) and is being used prospectively to develop combination vaccines for additional pathogens affecting swine. The EpiCC tool is the most recent tool to be integrated into iVAX; potential applications include determining where influenza virus “spillover” events will occur (transmission of influenza from swine to humans) and “predicting the next pandemic” in collaboration with influenza researchers at the University of Georgia, Athens, USA.

### Developing Safe and Effective Cell Therapies for Cancer

#### Predicting Adverse Effects of an Engineered TCR

**Figure 7:**
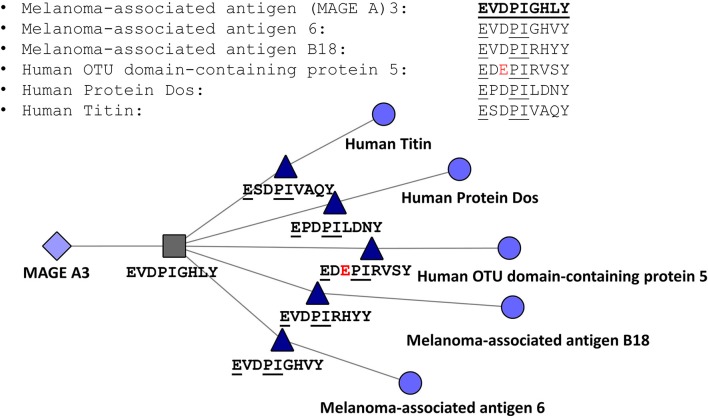
JanusMatrix Analysis of MageA3-HLA*0101-restricted T cell epitope. Peptides containing identical T cell facing residues include variants of MageA3 (Melanoma Associated Antigen) and three additional human proteins, including human titin, one of the components of cardiac smooth muscle. The figure shows that the HLA-A*0101-restricted T cell epitope EVDPIGHVY is conserved with an epitope that has identical HLA binding restriction but slightly different agretope (XSPDXXVAQY) and identical TCR facing residues (EXXPIXXXX).

The JanusMatrix tool may also be relevant for avoiding off-target effects of cell therapies, such as TCR-engineered T cells or CAR-TCR cells. To illustrate the potential application of JanusMatrix to searching for safety signals in cell therapy, we performed an entirely *in silico* approach to evaluate potential cross-reactivity of an engineered TCR directed at a MageA3 peptide presented by HLA A^*^0101 on tumors that was associated with significant (lethal) off target effects (66). JanusMatrix confirmed the potential for cross-reactivity at the TCR face of similar self-epitopes predicted to be presented by HLA A^*^0101. One of these epitopes was derived from Titin, a protein found in cardiac muscle. The off-target effect of this TCR-engineered T cell therapy was attributed in a separate report to its potential for targeting Titin epitopes [Figure 7; (67)]. Concern about off target effects of engineered T cells and cancer vaccines is leading to the development of additional algorithms that use T cell epitope comparison to evaluate cancer vaccine risk (68).

### Evaluating the Immunogenicity of CAR-T Transgenes

Autologous T cells that have been transduced with genes that express anti-tumor-specific antigens, such as CD19, have been demonstrated to have significant antitumor activity in B cell malignancies. However, immunogenicity may affect the efficacy of these T cells. In studies published by the CAR-T company Juno, CD8^+^ T cell–mediated anti-CAR transgene product immune responses developed after CAR–T cell infusion in some patients. These CD8 T cell responses limited CAR–T cell persistence and increased the risk of relapse risk. In the published study, five patients that had developed persistent leukemia or relapse after an initial infusion of anti-CD19 CAR-T received a second infusion of CAR-T cells (69). There was no expansion or persistence of CAR-T cells or demonstrable antitumor activity in any of the five patients and infusion was followed by the loss of CAR-T transduced T cell population.

A follow up study evaluated T cell responses to overlapping peptides *in vitro*. This publication showed that the VH and VL domains of the CAR-T had a high amount of Class I epitope content. Using ^*^A0101 and ^*^A0301 as proxies for the reported patient HLA type, EpiMatrix analysis showed agreement between predictions and immunogenic sequences. We note here that, apart from the scFv, there are other components that are part of the CAR-T structure (IgG4-hinge spacer, a CD28 transmembrane domain, a 4-1BB costimulatory domain, and a CD3ζ signaling domain). These sequences are human, but do have T cell epitope rich regions that potentially contribute to the reported immunogenicity of CAR-T.

## Conclusion

Advances in vaccine design using computational tools, measurement of vaccine efficacy using entirely *in vitro* systems, and vaccine delivery using a broad range of flexible platforms are moving vaccine science beyond traditional “shake and bake” (whole killed vaccines) of yesteryear to “forecast and tell” design methodologies of the future. Over the past 20 years, the teams working at EpiVax and affiliated academic institutions have developed tools to design vaccines and provided *in vitro* and *in vivo* evidence that immunoinformatics tools can be applied to vaccines that can effectively protect against live bacterial and viral challenge. These tools can also be used to improve the design of existing vaccines and to identify biomarkers of safety and efficacy. The effective integration of *in silico* immunoinformatics tools and *ex vivo*/*in vitro* and *in vivo* immune system technologies across the entire vaccine development pipeline will enable developers to predict and assess safety, toxicity, efficacy, quality, and performance of vaccines, accelerating the development of safe, and effective immunotherapy for a broad range of pathogens and species.

New tools described in this report have been validated in retrospective and prospective studies described briefly here (in anticipation of extended publication of the results elsewhere) and in previous publications. We have shown that EpiMatrix and JanusMatrix algorithms efficiently identify putative T cell epitopes, distinguish likely inflammatory peptides from potential regulatory peptides and are adaptable to a patient HLA-specific level of assessment. These results confirm that combining HLA-specific epitope content and “selfness” improves prediction of immunogenicity over either metric alone. Applying both tools in the early stages of vaccine design, antigen selection, and engineering is likely to result in the advancement of next generation vaccines where the minimal essential components of protection can be delivered without off-target or unintentionally suppressive signals deleterious to vaccine efficacy.

A number of commercial and academic teams are actively developing new vaccine design tools and have also contributed to innovation in the area of computational vaccinology. Several groups have for example developed public and private computational tools for predicting proteasomal cleavage sites [NetChop (70)], class I [netMHC (71), netMHCpan (72), MHCflurry (73), EDGE (74)], and class II [netMHCII and netMHCIIpan (75), TEPITOPE (76), RECON (77)] MHC-restricted T cell epitopes, as well as tools for identifying linear and discontinuous B cell epitopes [BepiPred (78), DiscoTope (79)].

The “iVAX toolkit” is currently in use by EpiVax, Inc. for clients that include commercial firms and academic collaborators developing novel vaccines for malaria, Q fever and Burkholderia for commercial development and biodefense contracts, and by academic groups that are engaged in federally funded programs for analyzing immune responses to emerging/re-emerging pathogens such as influenza (University of Georgia, Saint Louis University) and pertussis (Intravacc, Innatoss, VIC, and CDC). Even more recently, EpiVax has created a seamless pipeline for the design of personalized cancer vaccines that capitalizes on iVAX and its vaccine development and T cell immunology expertise called Ancer. Collaborative studies carried out using iVAX have demonstrated the importance of computational tools for vaccine design and vaccine efficacy studies. Additional collaborations to evaluate the potential efficacy of existing vaccines against newly emerging variants, to predict immune response to candidate antigens from newly emerging strains of infectious diseases, and to design novel vaccines that have enhanced immunogenicity at an accelerated pace are possible and encouraged.

## The Future

### Developing Personalized Vaccines

Traditional cancer vaccines based on Tumor Associated Antigens (TAAs) have for the most part failed in clinical trials (80–82). Evidence that is now emerging suggests that the reason they have failed is because TAAs are broadly recognized as “Self” by the immune system. New strategies are emerging in which these antigens may be modified to increase immunogenicity, using tools such as the ones described here. Careful analysis of some of the standard TAAs that have been used in clinical studies reveals the presence of putative Treg inducing epitopes that may be responsible for an immune-suppressive effect.

Meanwhile, the field of cancer therapy has undergone a major transformation in less than a decade due to the introduction of checkpoint inhibitors (CPI), next generation sequencing (NGS) and new emphasis on neo-epitopes. Neo-epitopes are T cell epitopes that contain tumor-specific non-synonymous amino acid mutations that effectively distinguish cancer (tumor) proteins from their normal counterparts. Neo-epitopes represent mutations to self-antigens that are recognized as “Non-Self” by the individual's own immune system, and are therefore capable of generating a potent immunogenic and clinical response. New strategies for developing personalized vaccines that direct immune responses to patient-specific T cell neo-epitopes are in development.

Unlike TAAs, cancer neo-epitopes are not found in normal tissue. Also, unlike TAAs that may be present in more than one tumor type and across patients, the vast majority of neo-epitopes are unique to each patient's tumor. The cost of sequencing individual tumor genomes to discover neo-epitopes dropped precipitously to a few thousand dollars per genome in 2018. When combined with CPI, therapeutic vaccines based on neo-epitopes are highly likely to improve outcomes and may well be effective as single agents in certain clinical settings.

Beginning in 2016, EpiVax developed an end-to-end immunoinformatics platform, separate from iVAX, that enables the rapid design of personalized, mutanome-directed therapeutic peptide cancer vaccines. These vaccines target multiple antigens specific to a patient's tumor, encoding several class I, and or class II HLA restricted neo-epitopes.

This proprietary immunoinformatics pipeline, which includes EpiMatrix and JanusMatrix and a version of iTEM called J-iTEM (see above descriptions) is called Ancer, and has been exclusively licensed to EpiVax Oncology, Inc., a subsidiary of EpiVax, established in 2017. EpiVax Oncology will use Ancer to develop individualized cancer vaccine by screening each cancer mutanome to identify tumor-specific neo-epitopes using EpiMatrix, filter out neo-epitopes that may be tolerogenic using JanusMatrix, and rank the remaining neoantigens according to their optimal immunogenic profile. While the number of companies that are developing tools for identifying neo-epitopes is rapidly expanding, having access to high throughput, commercial-grade and validated tools that incorporate consideration of the rules of self-tolerance will be critically important for the design of more effective and safer vaccines for cancer immunotherapy.

### The Next Frontier

The developers of the iVAX toolkit and the Ancer pipeline can imagine a not-to-distant future when many “immunotherapies”—not only for cancer, but also for infectious diseases, autoimmune disease, and allergies, can be designed and administered within weeks of diagnosis. We also anticipate broader application of the tools to pathogens afflicting food animals, including fish, and companion animals, as quickly as additional epitope prediction capacity can be added. Twenty years of computational development and collaborative validation studies have built a strong foundation. Computational vaccinology has a bright future and enormous potential to improve human and animal health.

## Data Availability Statement

The datasets analyzed in this article are not publicly available, as they are part of ongoing research and will be published at a later date. Requests to access the datasets should be directed to William Martin, martinb@epivax.com.

## Author Contributions

All authors listed have made a substantial, direct and intellectual contribution to the work, and approved it for publication.

### Conflict of Interest

AN and VK are employees of Leidos, Inc., the prime contractor for the Malaria Vaccine Development Program (MVDP), Contract AID-OAA-C-15-00071, with the Office of Infectious Diseases, Bureau for Global Health, U.S. Agency for International Development (USAID). AD and WM are senior officers and shareholders, and LM, FT, AG, PH, GR, and MA are employees of EpiVax, Inc., a privately owned biotechnology company located in Providence, RI. These authors acknowledge that there is a potential conflict of interest related to their relationship with EpiVax and attest that the work contained in this research report is free of any bias that might be associated with the commercial goals of the company. The remaining authors declare that the research was conducted in the absence of any commercial or financial relationships that could be construed as a potential conflict of interest.
